# Effect of Spirulina and Fish Processing By-Products Extracts on Citrinin-Induced Cytotoxicity in SH-SY5Y Cells

**DOI:** 10.3390/foods13121932

**Published:** 2024-06-19

**Authors:** Francisco J. Martí-Quijal, Juan Manuel Castagnini, Francisco J. Barba, María José Ruiz

**Affiliations:** 1 Research Group in Innovative Technologies for Sustainable Food (ALISOST), Food Chemistry and Toxicology Laboratory, Preventive Medicine and Public Health, Food Science, Toxicology and Forensic Medicine Department, Faculty of Pharmacy, Universitat de València, Avda. Vicent Andrés Estellés, s/n, 46100 Burjassot, València, Spain; francisco.j.marti@uv.es (F.J.M.-Q.); francisco.barba@uv.es (F.J.B.); 2 Research Group in Alternative Methods for Determining Toxics Effects and Risk Assessment of Contaminants and Mixtures (RiskTox), Food Chemistry and Toxicology Laboratory, Preventive Medicine and Public Health, Food Science, Toxicology and Forensic Medicine Department, Faculty of Pharmacy, Universitat de València, Avda. Vicent Andrés Estellés, s/n, 46100 Burjassot, València, Spain

**Keywords:** citrinin, spirulina (*Arthrospira platensis*), fish by-products, SH-SY5Y, sea bass head

## Abstract

Citrinin (CIT) is a mycotoxin commonly found in grains, fruits, herbs, and spices. Its toxicity primarily affects the kidney and liver. Meanwhile, food industry by-products, particularly from fishing and aquaculture, contribute significantly to environmental concerns but can also serve as valuable sources of nutrients and bioactive compounds. Additionally, microalgae like spirulina (*Arthrospira platensis*) offer interesting high-added-value compounds with potential biological and cytoprotective properties. This study aims to reduce CIT’s toxicity on SH-SY5Y cells using natural extracts from the microalgae spirulina and fish processing by-products (sea bass head). The combination of these extracts with CIT has shown increased cell viability up to 15% for fish by-products extract and about 10% for spirulina extract compared to CIT alone. Furthermore, a notable reduction of up to 63.2% in apoptosis has been observed when fish by-products extracts were combined with CIT, counteracting the effects of CIT alone. However, the extracts’ effectiveness in preventing CIT toxicity in the cell cycle remains unclear. Overall, considering these nutrient and bioactive compound sources is crucial for enhancing food safety and mitigating the harmful effects of contaminants such as mycotoxins. Nevertheless, further studies are needed to investigate their mechanisms of action and better understand their protective effects more comprehensively.

## 1. Introduction

Mycotoxins, toxic metabolites produced by certain fungi, have become increasingly relevant in scientific, health, and food contexts. These compounds can contaminate various agricultural products such as cereals, fruits, nuts, and processed foods, becoming a significant threat to food safety and human health [1,2].

In particular, citrinin (CIT), a mycotoxin produced by species of *Aspergillus*, *Penicillium*, and *Monascus* genera, has raised growing concerns due to its toxicity and occurrence in food and feed [3,4]. CIT has been associated with adverse effects on the renal and hepatic systems, and its presence in grain-derived and fermented products presents considerable challenges for the food industry and regulatory agencies in terms of contamination control and prevention [5]. Citrinin has been detected in foods and food supplements in a range from 0.10 to 44,240 µg/kg [6]. In the case of human exposure, there is no established limit except for food supplements (red rice yeast). Therefore, the available information on dietary exposure to citrinin is still scarce. In comparison to the tolerable daily intake (TDI) of 0.2 μg/kg body weight/day, which corresponds to the ‘nephrotoxicity level of no concern’ established by the European Food Safety Authority (EFSA) in 2012 [7], Ali and Degen [8] reported that human exposure ranged from 1.8 ± 1.1% to 103.3 ± 244.1% of TDI, depending on the studied population.

In addition to mycotoxins, the generation of agri-food industry by-products is a critical issue of concern. The production and processing of food result in the generation of by-products such as fruit peels, animal bones, and processing residues. These by-products can present both environmental and economic challenges, but they also hold significant potential for obtaining valuable nutrients and bioactive compounds, including antioxidants, bioactive peptides, and enzymes [9].

Among the most relevant food industry by-products, those originating from the fishery industry deserve special attention. Fishery by-products, such as heads, backbones, skin, and viscera, represent a rich source of nutrients and bioactive compounds, including peptides, oils, collagen, and minerals, which can find applications in functional foods and pharmaceutical products [10,11,12,13,14,15,16]. Harnessing these by-products sustainably and efficiently can contribute to the valorisation of the fishery industry and the reduction of environmental impact [17,18,19].

In addition, in the quest for new sources of nutrients and bioactive compounds, microalgae have emerged as a promising alternative. In particular, spirulina, a green-blue microalga with high nutritional value, has stood out for its exceptional contents of proteins, vitamins, minerals, and antioxidant compounds [20,21,22]. Spirulina has demonstrated a wide range of health benefits, including antioxidant, anti-inflammatory, and anticancer properties, and has become a key resource in the formulation of functional foods and nutritional supplements [23,24].

While existing literature has utilized various cell lines, e.g.,: mouse Sertoli cell line (TM4), human hepatocarcinoma cell lines (HepG2 and Hep3B), Chinese hamster lung fibroblasts (V79) cells, porcine kidney epithelial (PK15) cells, or mouse skeletal muscle (C2C12) cells [25,26,27,28,29,30,31], to assess CIT toxicity (e.g., IC_50_ studies), little evidence exists on the mechanisms of CIT toxicity in SH-SY5Y cells and the potential use of natural compounds to mitigate these effects. To contribute new insights to the scientific literature, we selected the SH-SY5Y line. Moreover, mechanistic toxicology to determine how substances disrupt biological targets are general processes applicable to any type of cell. Therefore, we believe employing this cell line can enhance the overall understanding of CIT’s toxic effects.

The objective of this study was to examine the impact of combining the mycotoxin CIT with two different natural extracts, one derived from fish by-products and the other from the microalgae spirulina, to mitigate the toxic effects induced by this mycotoxin by in vitro methods. The research will assess cell viability using the MTT assay, alongside other cellular processes including cell cycle and apoptosis by flow cytometry. To determine the cytoprotective effects of the extracts obtained, a comparative analysis will be conducted between the effects of CIT tested alone in a cell culture model and CIT in combination with each of the extracts proposed.

The novelty of this study resides, on one hand, in utilizing a cell line that has received limited attention in toxicological studies investigating the mycotoxin CIT. On the other hand, it involves the use of extracts of marine origin, with one derived from fish by-products using innovative, green, and sustainable extraction approaches—specifically, pulsed electric fields (PEF). This approach not only broadens the exploration of the cell line’s applicability but also investigates the potential of these by-products as strategies to mitigate the toxicity induced by mycotoxins.

## 2. Materials and Methods

### 2.1. Reagents

Sigma Chemical Co (St. Louis, MO, USA) provided the following reagents: culture medium DMEM Ham’s-F12, trypsin/EDTA solutions, fetal bovine serum (FBS), penicillin, streptomycin, phosphate buffer saline (PBS), Ribonuclease A (RNase), tetrazolium bromide (MTT), trizma base (Tris), propidium iodide (PI), t-octylphenoxypolyethoxyethanol (Triton-X 100), and HEPES. Human recombinant annexin V-FITC conjugate was obtained from Invitrogen (Carlsbad, CA, USA). Merck KGaA (Darmstadt, Germany) supplied NaCl. The citrinin (CIT) standard was also purchased from Sigma-Aldrich (St. Louis, MO, USA), and dimethyl sulfoxide (DMSO) was acquired from Fisher Scientific (Geel, Belgium). CIT standard was resuspended in pure DMSO.

### 2.2. Samples

The sea bass head PEF extract (HPO) was obtained following the method described by Martí-Quijal et al. [32]. Briefly, the sea bass head was treated by PEF, using the following parameters: a specific energy of 220 kJ/kg, a field strength of 1 kV/cm, a pulse duration of 100 ms, and a frequency of 2 Hz, with a unipolar square wave pulse. Then, it was stirred at 200 rpm for 21.35 h in water. Finally, the sample was centrifuged at 3050× *g* for 10 min in a 5810R centrifuge (Eppendorf AG, Hamburg, Germany) and the supernatant was collected. The extract was characterized in a previous work of our research group [32]. The extract was used at 8.25% and 12.5% (*v*/*v*) of total extract in the cell culture medium.

In addition, spirulina (*Arthrospira platensis*) ethanolic extract (SpEe) was obtained as reported by Sansone et al. in a previous article [33]. In summary, a total of 5 g (dry weight) of microalgae were placed into a suspension with 50 mL of pure ethanol and subjected to agitation in the absence of light at 500 rpm for 30 min. Subsequently, the resultant mixture underwent separation through centrifugation at 3050× *g* for 10 min, with the resulting liquid phase being carefully transferred into a clean tube. The remaining pellet was then resuspended in 50 mL of ethanol and subjected to a second round of extraction. Following the repetition of the separation step via centrifugation, the extracts from both rounds were combined. The final extract was characterized in a previous work of our research group [34]. The extract was dried and resuspended in DMSO to obtain the final concentrations used in the present work (31.25 µg/mL and 62.5 µg/mL of extract dry weight in cell culture medium).

### 2.3. Cell Culture

Human neuroblastoma (SH-SY5Y) cells obtained from American Type Culture Collection (ATCC CRL-2266) were cultured in Dulbecco’s Modified Eagle Medium (DMEM) Ham’s-F12 medium enriched with 10% fetal bovine serum (FBS), 100 mg/mL streptomycin, and 100 U/mL penicillin. The cells were maintained under standard conditions at 37 °C, 5% CO_2_, and a pH of 7.4 in a 95% air atmosphere with constant humidity. The culture medium was changed every 2–3 days. To attain the desired concentrations of CIT, CIT + SpEe, and CIT + HPO, appropriate solutions were prepared and added to the culture medium, ensuring a final DMSO concentration of ≤1% (*v*/*v*). The concentrations selected for CIT (25, 38.75, and 50 µM) were fixed from the IC_50_ of CIT measured by MTT (75 µM): IC_50_/1.5 ≈ 50 µM; IC_50_/2 ≈ 38.75 µM; IC_50_/3 ≈ 25 µM). Control groups, containing equivalent amounts of solvent (DMSO), were included in all experiments.

### 2.4. Cell Viability Assay

The cell viability was determined using the MTT assay [35,36]. For that purpose, SH-SY5Y cells were seeded in 96 well plates at a density of 30,000 cells/well. Once the cells reached 80% of confluence, CIT at different concentrations with or without the different extracts tested (SpEe or HPO) was added. After 24 h of exposure, the supernatant was removed and 200 µL of fresh medium with 50 µL of MTT (5 mg/mL) were added. Then, the cells were incubated at 37 °C for 3 h in darkness. Finally, the supernatant was removed and DMSO was added to solubilize the formazan crystals. Absorbance was read at 630 nm using a VICTOR3 1420 multilabel plate counter reader (PerkinElmer, Turku, Finland).

### 2.5. Cell Cycle Analysis

The effects on the SH-SY5Y cell cycle were determined by flow cytometry as previously described by Zingales et al. [37], using Vindelov’s PI staining reagent. For that purpose, 700,000 cells/well were seeded in 6 well plates. Then, after 24 h, the cells were exposed to the different concentrations of CIT (25, 38.75, and 50 µM) both with SpEe or HPO extracts and without them. After 24 h of exposure, cells were trypsinized and incubated at 4 °C for 30 min in darkness with 500 µL of Vindelov’s PI staining solution. Finally, 20,000 events were analysed for each sample in a BD LSRFortessa (BDBiosciences, Franklin Lakes, NJ, USA) flow cytometer. The Vindelov’s PI staining reagent was prepared by mixing 10 mM Tris, 0.1% Triton X-100, 40 µg/mL RNase, 50 µg/mL of PI, and 10 mM NaCl in PBS. Three independent experiments were performed.

### 2.6. Apoptosis Measurement

The apoptosis process in SH-SY5Y cells was performed following the protocol described by Zingales et al. [38]. The double stain Annexin V-FITC/PI was used for this purpose. To carry out the experiment, 700,000 cells/well were seeded in 6 well plates and, after 24 h, they were exposed to the different concentrations of citrinin (25, 38.75, and 50 µM) both with SpEe or HPO extracts and without them. After 24 h of exposure, cells were trypsinized and resuspended in 500 µL of Hepes-Ca^2+^ buffer containing Annexin V-FITC/PI. Finally, 10,000 events were acquired and analysed by a BD LSRFortessa (BD Biosciensces, Franklin Lakes, NJ, USA) flow cytometer. Early apoptotic (Annexin V-FITC+/PI−) and late apoptotic (Annexin V-FITC+/PI+) cells were evaluated from the total population of cells. Three independent experiments were performed.

### 2.7. Statistical Analysis

The statistical analysis was performed using the software GraphPad Prism 9 (GraphPad Software, San Diego, CA, USA). Results were expressed as Mean ± SEM of three independent experiments. The statistical analysis of the results was carried out using a 2-way ANOVA followed by Tukey’s multiple comparisons test. Differences were considered statistically significant when *p* ≤ 0.05.

## 3. Results and Discussion

### 3.1. Effect of Spirulina and Fish By-Products Extracts on Cell Viability

#### 3.1.1. Fish By-Products Extracts

Extracts obtained from sea bass by-products (head, skin, viscera, and backbones) by agitation (control) and pre-treatment with PEF were characterized in a previous study and evaluated on SH-SY5Y cells at concentrations ranging from 0.78% to 25% (*v*/*v*) by MTT test at 24 h of exposure [32].

After assessing each side stream individually at 24 h of exposure, it was found that the head extract obtained by PEF (HPO) increased cell viability by 25% at a concentration of 8.25% (*v*/*v* in medium) and by 33% at a concentration of 12.5% (*v*/*v* in medium). However, both the skin extract obtained by PEF and the skin extract obtained by agitation (control) showed a significant decrease in cell viability. In contrast, as observed in a previous study developed in our laboratory, the extracts of backbone and viscera did not exhibit any significant changes when compared to the control [32].

Regarding the composition of the HPO extract, it was rich in protein, with a concentration of 28.92 ± 3.22 g/100 mL. In addition, it contained several bioactive peptides with potential antioxidant activity. Among the minerals present in the extract were 8.7 ± 0.1 µg/L magnesium (Mg), 52.1 ± 1.2 µg/L phosphorus (P), 12.7 ± 0.5 µg/L calcium (Ca), 0.326 ± 0.006 µg/L iron (Fe), and 0.402 ± 0.005 µg/L zinc (Zn) [32].

Based on these results, the HPO extract was selected for future experiments as it had the highest content of antioxidant compounds and showed the best response in terms of cell viability. Concentrations of 8.25% (*v*/*v*) and 12.5% (*v*/*v*) were chosen to be combined in further experiments with the mycotoxin, as they produced the most promising results on cell viability.

#### 3.1.2. Spirulina Ethanolic Extract

The spirulina (*Arthrospira platensis*) ethanolic extract (SpEe) was obtained by agitation with ethanol. It was characterized in a previous study [34]. The SpEe contained a variety of bioactive compounds and essential nutrients. Among the pigments, 2.838 ± 0.081 mg/g dry matter (DM) of phycocyanin, 5.612 ± 0.547 mg/g DM of chlorophyll a, and 0.447 ± 0.096 mg/g DM of carotenoids were found. Concerning minerals, the extract contained 49.33 ± 1.00 μg/g DM of Mg, 52.98 ± 2.86 μg/g DM of P, 52.27 ± 1.43 μg/g DM of Ca, and 0.87 ± 0.04 μg/g DM of Fe. The fatty acids present included 3.056 ± 0.100 mg/g DM of γ-linolenic acid, 0.941 ± 0.038 mg/g DM of palmitoleic acid, 0.200 ± 0.007 mg/g DM of stearic acid, 0.037 ± 0.001 mg/g DM of eicosadienoic acid, 0.024 ± 0.001 mg/g DM of eicosatrienoic acid, and 2.200 ± 0.060 mg/g DM of linoleic acid. In addition, the phenolic profile of the extract includes hydroxybenzoic acid, 4-hydroxybenzaldehyde, protocatechuic acid, hesperidin, and apigenin [34].

The concentrations chosen for combining the SpEe with CIT were 31.25 µg/mL and 62.5 µg/mL based on previous tests performed in our laboratory. It should be noted that at the concentrations selected, the SpEe extract did not exhibit any reduction in cell viability [34].

### 3.2. Cytotoxic Effects Concerning the Combination of Natural Extracts with Citrinin

#### 3.2.1. HPO Extract in Combination with Citrinin

**Figure 1:**
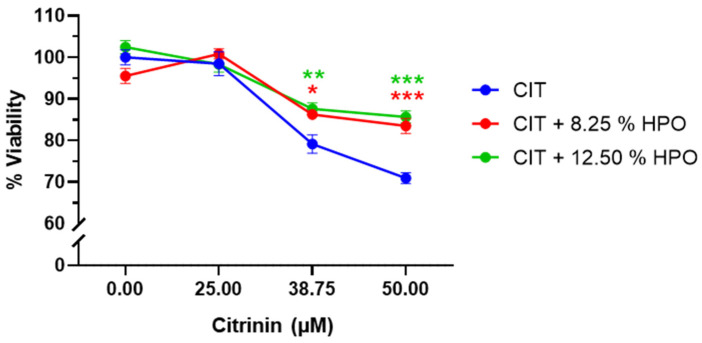
Effect on cell viability of CIT, and CIT with Head PEF Opt. extract (HPO) at two different concentrations (8.25 and 12.5% (*v*/*v*)). The evaluated concentrations of CIT were 25, 38.75, and 50 µM. The results are presented as Mean ± SEM of three independent experiments. * *p* < 0.05; ** *p* < 0.01; *** *p* < 0.001 vs. CIT (alone). A 2-way ANOVA followed by Tukey’s multiple comparisons test was performed. CIT: Citrinin; HPO: Head PEF Opt. extract. Control groups for the extracts without toxin (containing equivalent concentrations of solvent) were shown as “Control” in the figure.

Figure 1 presents the results obtained for cell viability following the combination of HPO extract at two different concentrations: 8.25% and 12.5% (*v*/*v*). At the lowest tested concentration of CIT, 25 µM, no significant differences were observed among CIT tested individually and in combination with the HPO extracts. However, a clear protective effect is evident when the HPO extracts (both concentrations) were combined with 38.75 µM and 50 µM CIT. When applied at a concentration of 38.75 µM CIT, cell viability increased from 79.2% to 86.3% (8.25% HPO) and 87.6% (12.5% HPO). It is noteworthy that at 50 µM CIT, the simultaneous use of the HPO extract increases viability by 15%, reaching 83.5% (8.25% HPO) and 85.6% (12.5% HPO), compared to the 70.9% viability observed with CIT alone. It is also important to highlight that no significant differences were observed between the two HPO tested concentrations. Thus, the lowest concentration (8.25% HPO) was selected for the subsequent toxicological studies.

This cytoprotective effect may be attributed to the action of different compounds present in fish extracts, such as bioactive peptides, among other nutrients. Some of these compounds also exhibit potential antioxidant activity and can help to improve cell viability [39,40,41,42,43].

These findings are in agreement with other studies reported in the literature. For example, Taroncher et al. [44] observed that hydrolysates from fish by-products, specifically salmon backbones and salmon viscera, increased cell viability by 27.0% and 51.2%, respectively, by MTT assay. In addition, the same authors reported higher increases in cell viability measured by total protein content assay. Cell viability increased 214% in mackerel head hydrolysate, compared to control, followed by salmon head and herring viscera hydrolysates, which achieved cell viability values of 140% and 139% compared to control (100%), respectively. These results indicate that hydrolysate exposure enhanced cell viability in Caco-2/TC7 cells. Moreover, it has been demonstrated that these extracts possess antioxidant capacity, because they contributed to the reduction of oxidative stress caused by reactive oxygen species in Caco-2/TC7 cells [44].

Additionally, it has been also demonstrated that fish extracts can enhance the antioxidant activity of natural antioxidant compounds. Concerning this, Taroncher et al. [45] demonstrated that cell proliferation was enhanced by the combination of vitamin E or resveratrol with hydrolysed protein from salmon fish. Similarly, another mixture containing hydrolysed protein from both mackerel and salmon fish blended with quercetin and vitamin C also demonstrated an increased cell proliferation.

On the other hand, Shashikumar et al. [46] observed that primary hepatocytes, which had been damaged by D-galactosamine, facilitated the restoration of their functions when exposed to fish oil. These functions are the production of albumin and the regulation of various liver enzymes (lactate dehydrogenase (LDH), alkaline phosphatase (ALP), glutamic-oxaloacetic transaminase (GOT), and glutamic pyruvic transaminase (GPT)), altered by the hepatotoxic injury. The recovery functions were dependent on the fish oil concentration, and the protective effect was observed at 10 and 20 µg/mL of oil.

Finally, Omerovic et al. [47] reported that the administration of an extract obtained from cod muscle decreased the mortality of mice treated with the anticancer drug doxorubicin, likely due to cardioprotection through antioxidant activity.

#### 3.2.2. Spirulina Ethanolic Extract (SpEe) in Combination with Citrinin

**Figure 2:**
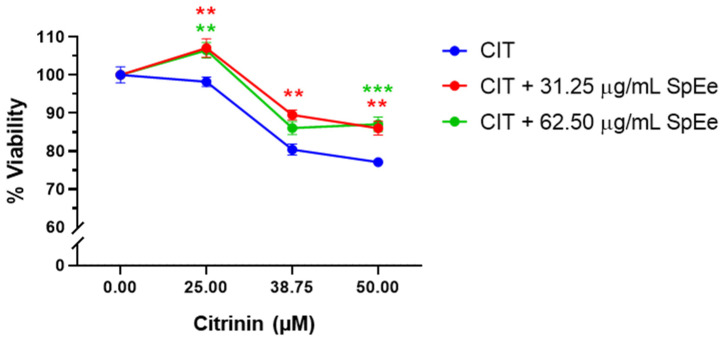
Effect of CIT (25, 38.75 and 50 µM), and CIT with ethanolic spirulina extract (SpEe) (31.25 and 62.5 µg/mL) on SH-SY5Y cell viability. The results are presented as Mean ± SEM of three independent experiments. ** *p* < 0.01; *** *p* < 0.001 vs. CIT tested individually. A 2-way ANOVA followed by Tukey’s multiple comparisons test was performed. CIT: Citrinin; SpEe.: ethanolic spirulina extract. Control groups for the extracts without toxin (containing equivalent concentrations of solvent) were shown as “Control” in the figure.

Figure 2 shows the results of cell viability obtained from the SH-SY5Y cells exposed to the combination of SpEe and CIT. Concentrations of 31.25 µg/mL and 62.5 µg/mL of SpEe were selected, and 25 µM, 38.75 µM, and 50 µM were tested for CIT. The results demonstrate the remarkable effectiveness of the SpEe in protecting cells against CIT cytotoxicity. Even at the lowest concentration tested (25 µM CIT), an increase in cell viability was observed with both SpEe concentrations, resulting in viability values of 107.1% (31.25 µg/mL SpEe) and 106.5% (62.5 µg/mL SpEe), compared to control (98.22%). The highest increase in cell viability was observed at the concentration of 38.75 µM CIT in combination with SpEe 31.25 µg/mL, reaching 89.5%; while the extract at 62.5 µg/mL only achieves 86.1% viability, which is not significantly different from the viability observed with the mycotoxin alone (80.4%). Furthermore, at 50 µM CIT, both extracts exhibit similar results, significantly increasing cell viability from 77.1% (CIT) to 87.0% (CIT + 31.25 µg/mL SpEe) and 86.0% (CIT + 62.5 µg/mL SpEe).

Other researchers have also reported the protective effect of microalgae extracts against toxins exposure in cell cultures. In this context, Kang et al. [48] reported a protective effect of a hydrolysate from the microalga *Navicula incerta* against damage caused by a 1-h exposure to 1 M ethanol in HepG2/CYP2E1 cells (HepG2 cells transfected with human CYP2E1 cDNA). For instance, these authors described that after a 48-h exposure to the hydrolysate, a protective effect was observed against a subsequent 48-h exposure to 1 M ethanol. It was due to enhanced mitochondrial antioxidant activity and improved functionality, owing to the components present in this protein hydrolysate.

On the other hand, Ben Saad et al. [49] described a protective effect of an ethanolic extract from the red algae *Alsidium corallinum* against potassium bromate-induced toxicity in mice. These authors observed that the algae extract helped the mice regain body weight compared to those who were only administered with the toxicant, resulting in weight loss. However, it was hypothesized that the weight gain was due to the minerals contained in the extract (elevated levels of Ca, Mg, Fe, Cu, and Zn). Additionally, the administration of the *Alsidium corallinum* extract led to the restoration of normal haematological parameters, such as haematocrit, haemoglobin, platelet, and red blood cell count, which were altered by exposure to KBrO_3_. The protective effect could be attributed to the presence of flavonoids in the extract, which prevent membrane fragility [49].

As evidenced in a previous study carried out in our laboratory, the selected SpEe of this research is rich in antioxidant compounds such as polyphenols and pigments like phycocyanin. Additionally, it contains fatty acids (including γ-linolenic acid, eicosadienoic acid, and eicosatrienoic acid), as well as essential minerals such as Mg, P, Ca, Fe, and Zn. Thus, according to the results, these constituents may account for the observed cytoprotective effect against CIT exposure in SH-SY5Y cells [34].

### 3.3. Effect of Natural Extracts Combination with Citrinin Regarding Cell Cycle

Cell-cycle analysis was conducted following the protocol described by Zingales et al. [37], employing Vindelov’s PI staining solution. This fluorescent dye can intercalate with double-stranded nucleic acids, allowing for accurate and efficient assessment of cellular DNA content through flow cytometric analysis.

**Figure 3:**
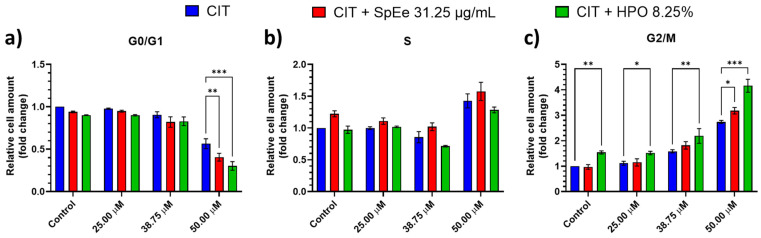
Flow cytometry results for cell cycle being (**a**) G0/G1 phase; (**b**) S phase and (**c**) G2/M phase of SH-SY5Y cells after 24 h of exposure to CIT, CIT + SpEe and CIT + HPO. Results are expressed as Mean ± SEM of three independent experiments. * *p* < 0.05; ** *p* < 0.01; *** *p* < 0.001 vs. CIT. A 2-way ANOVA followed by Tukey’s multiple comparisons test was performed. CIT: Citrinin; HPO: Head PEF Opt. extract; SpEe.: ethanolic spirulina extract. Control groups for the extracts without toxin (containing equivalent concentrations of solvent) were shown as “Control” in the figure.

Figure 3 describes the results obtained for cell cycle analysis in SH-SY5Y cells after 24 h of exposure to CIT, CIT + SpEe and CIT + HPO. As can be seen, CIT alone produces a concentration-dependent increase of cells in S-phase and especially in G2/M compared to the control condition, with 50 µM CIT being the condition with the greatest increase in both cases (an increase of 0.58 and 1.74 times for S and G2/M phase, respectively, compared to CIT control). Concerning the combination of CIT with the extracts, as can be observed, during the G2/M phase, there is a higher proportion of cells in this phase in the presence of the CIT + HPO combination compared to CIT alone (in a range from 0.40 to 1.42 times more, compared to each concentration of CIT alone), across all tested conditions (Control, 25, 38.75, and 50 µM) (Figure 3c). The fact that this increase is already observed in the HPO Control conditions (without CIT) raises suspicions that it may be a result of enhanced cell proliferation facilitated by the HPO extract. Indeed, Huang et al. [50] observed similar outcomes when exposing HaCaT cells to gelatines extracted from extrusion-treated tilapia fish scales. Their findings revealed a reduction in the number of cells in the G0/G1 phase and a concurrent rise in the proportion of cells in the G2/M phase compared to the control group, which is the same behaviour as described in our study. Those authors suggested that the heightened proliferation rate can be attributed to an increased frequency of entry into the S-phase, signifying that a substantial portion of the cell population is actively undergoing mitosis. Therefore, this increase in cells in the G2/M phase, resulting from enhanced cell proliferation in cells exposed to fish extract (already present in the control condition), adds to the G2/M phase block induced by the CIT mycotoxin [51], demonstrating an additive effect. However, as described earlier, the HPO extract exerts a protective effect on SH-SY5Y cells, reducing CIT cytotoxicity and thereby increasing cell viability. Consequently, further studies will be necessary to delve into the implications that the co-exposure of CIT and HPO has on the cell cycle effects of SH-SY5Y cells.

Regarding the S phase, no significant differences were observed among the tested conditions. In fact, the behaviour remains consistent across all exposure conditions, that is CIT alone or in combination with the extracts.

Lastly, as shown in Figure 3a, a significant reduction in the proportion of cells in the G0/G1 phase at the highest concentration of 50 µM CIT was observed. There is a reduction in this phase, both in the SpEe (*p* < 0.01) and in the HPO extract (*p* < 0.001) compared to 50 µM CIT tested alone. Similar results were obtained by Huang et al. [50], which has been discussed above.

Therefore, it can be concluded that none of the tested extracts induces relevant changes in the effect of the CIT mycotoxin on the cell cycle in SH-SY5Y cells.

The extracts studied in this research (HPO and SpEe) did not demonstrate a cytoprotective effect on the toxic effect of CIT at a cell cycle level. There are different works in the literature that use extracts obtained from marine biomass to mitigate the harmful effects of different toxins on the cell cycle function. For instance, Yang et al. [52] observed that the addition of an aqueous extract of the seaweed *Gracilaria tenuistipitata* (AEGT) protected H1299 cells from damage caused by 24 h of exposure to H_2_O_2_. Specifically, the authors noted that the extract reversed the G2/M phase arrest induced by H_2_O_2_ in H1299 cells when it was combined with 4 mg/mL of AEGT, achieving similar results to the control cells.

Furthermore, Lee et al. [53] reported a cytoprotective effect when applying an ethanolic extract of spirulina (*A. platensis*) to nHDF cells after UVB irradiation. They observed an increase in S and G2/M phases (1.7 fold and 1.3 fold, respectively, compared to the control). These authors found that the highest concentration of the extract (20 μg/mL) during the UVB radiation exposure reduced cell population values for the G1/G0 phase (74.23%), while increased for the S phase (8.99%), and for the G2/M phase (11.81%) with respect to the control (79.11%, 6.89%, and 10.50% for the G0/G1, S, and G2/M phases, respectively).

The differences between the results obtained in our study and those reported by Lee et al. may be attributed to the different sources of cellular damage, and the type of cells employed. Lee et al. used human dermal fibroblasts (nHDFs) and Yang et al. used human lung adenocarcinoma cells (H1299), whereas we utilized SH-SY5Y cells, which are a neuroblastoma tumour cell line.

### 3.4. Effect of Natural Extracts Combination with Citrinin Regarding Apoptosis Process

Cell death can occur through necrosis or apoptosis. During apoptosis, phosphatidylserine (PS) is externalized to the outer membrane. Annexin V-FITC/PI double staining is used to distinguish between apoptotic (early and late) and necrotic cell populations. Annexin V, a calcium-dependent phospholipid-binding protein, binds to cells displaying externalized PS, while PI binds to the DNA of necrotic or dead cells. Viable cells exclude PI, early apoptotic cells are Annexin V-FITC positive, and late apoptotic cells, transitioning to necrosis, are both Annexin V-FITC and PI positive [54]. This staining technique helps to identify and differentiate these cell states based on their membrane integrity and apoptotic progression.

**Figure 4:**
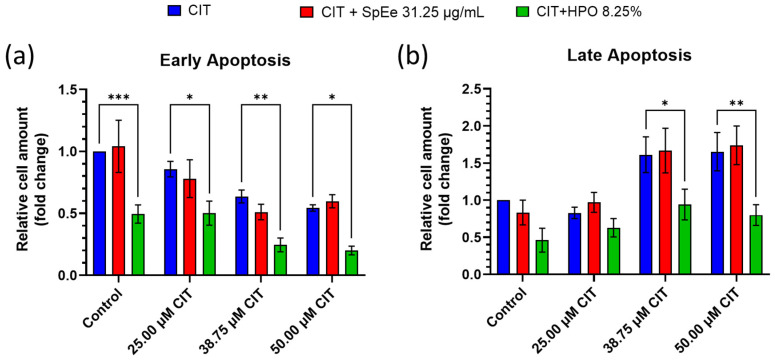
(**a**) Early and (**b**) Late apoptosis induction in SH-SY5Y cells after exposure to CIT, CIT + SpEe and CIT + HPO for 24 h. Results are expressed as Mean ± SEM of three independent experiments. * *p* < 0.05; ** *p* < 0.01; *** *p* < 0.001 vs. CIT. A 2-way ANOVA followed by Tukey’s multiple comparisons test was performed. CIT: Citrinin; HPO: Head PEF Opt. extract; SpEe: ethanolic spirulina extract. Control groups for the extracts without toxin (containing equivalent concentrations of solvent) were shown as “Control” in the figure.

The effect of the mycotoxin CIT and its combination with SpEe and HPO on apoptosis is depicted in Figure 4. As can be observed, there is a significant reduction in apoptotic cells in a concentration-dependent manner for all treatments, CIT and its combination with the extracts. The highest decrease in early apoptotic cells was observed when CIT was combined with the HPO extract compared to CIT tested alone (Figure 4a). In particular, early apoptotic cell population decreased to 50.6%, 41.5%, 61.5%, and 63.2% for Control (0 µM CIT), 25, 38.75, and 50 µM CIT, respectively. It is noteworthy that even in the control sample, the HPO extract alone demonstrates the ability to decrease the apoptotic cell population, potentially linked to the higher cell viability observed associated with this extract. Additionally, a significant decrease in early apoptosis is observed even at the highest concentration tested (50 µM), from 0.54 (50 µM CIT) to 0.20 (50 µM CIT + HPO) folds, indicating a protective capacity of the extract even under high mycotoxin concentrations.

On the other hand, regarding late apoptosis, Figure 4b shows late apoptotic of CIT and its extract combination after 24 h of exposure in SH-SY5Y cells. Differences between the addition of HPO extract and the CIT tested alone are only observed at the two highest concentrations tested, 38.75 and 50 µM. The value of late apoptosis is approximately half of that obtained with CIT alone, decreasing from 1.61 to 0.94 folds compared to the CIT tested alone for 38.75 µM and from 1.65 to 0.79 for 50 µM CIT tested alone. The anti-apoptotic activity demonstrated by HPO extract may be attributed to the presence of peptides that could interfere with the apoptosis process induced by the CIT mycotoxin.

On the contrary, there were no changes observed in either the early apoptotic or the late apoptotic cells when comparing SH-SY5Y cells exposed to CIT + SpEe with the ones exposed to CIT alone at any of the CIT concentrations tested. Therefore, this result suggests that the mechanisms through which SH-SY5Y cell viability is higher in CIT + SpEe than in CIT alone exposed cells are unrelated to the apoptotic process.

The results obtained in this study are consistent with the findings reported in other studies in the available literature. Remarkably, Gómez et al. [55] evaluated various cytoprotective properties of a hydrolysate of red tilapia (*Oreochromis* spp.) viscera in Caco-2 cells. The authors found that pre-treatment with the smallest peptide fraction with a molecular weight below 1 kDa (isolated by ultrafiltration from the whole hydrolysate) before applying 5 mM of H_2_O_2_ resulted in a 28.8% reduction in late apoptotic cells compared to cells exposed only to H_2_O_2_, achieving values similar to the control condition.

The research conducted by Gao et al. [56] investigated the protective effect of a hydrolysate derived from tilapia by-products, specifically from the skin. The study revealed that the addition of tilapia skin peptides at a concentration of 50 ng/mL provided protection to CT-26 cells against apoptosis induced by the addition of 1 μg/mL lipopolysaccharide (LPS). This effect was also observed in HT-29 cells.

Similar results were described by Hoon Song et al. [57]. For instance, these authors studied the impact of fish collagen peptides (FCP) on mouse thymic cortical epithelial reticular cells exposed to cisplatin (a commonly used chemotherapy drug). They found that cisplatin treatment changed proteins associated with the apoptotic process, causing a decrease in the expression of anti-apoptotic proteins and an increase in the expression of pro-apoptotic proteins. However, pre-treatment with 0.08% FCP for 24 h mitigated these changes, restoring the expression of apoptosis-related proteins closer to their normal levels. FCP demonstrated the ability to enhance the expression of certain protective proteins while reducing the levels of harmful ones when compared to cells treated with cisplatin alone.

## 4. Conclusions

The results of this study demonstrate the promising cytoprotective effects of the extracts obtained from fish by-products, specifically sea bass head, and spirulina microalgae in reducing the cytotoxicity of CIT in SH-SY5Y cells by improving cell viability and decreasing apoptosis. Additionally, the spirulina extract showed the potential to increase cell viability when combined with CIT. Furthermore, it was observed that the protective effect of both extracts was obtained at all concentrations tested.

The application of the obtained extracts, both fish by-products and microalgae, contributes to the development of a sustainable protocol that can help companies reduce the cost and environmental impact of waste from raw materials and improve circular economy. Then, this study stands as a pivotal contribution to the arsenal of mitigation approaches, paving the way for a more sustainable future. However, taking into account the 2-way approach followed, on the one hand, the investigation of alternative sources (i.e., fish by-products and spirulina extracts) and innovative extraction technologies (PEF) in cytoprotection, further exploration and in-depth investigations are needed to fully harness the potential of the biomasses and technologies as well as to elucidate the underlying mechanism of the cytoprotective effect of spirulina and fish by-products extracts. Based on the results obtained in this study, future research should focus on further analysis of the molecular mechanisms underlying the cytoprotective effects of fish by-product extracts and spirulina. In particular, it would be valuable to examine how these extracts affect oxidative stress and inflammatory pathways in cells exposed to citrinin. This can be achieved by assessing the impact of the extracts on reactive oxygen species (ROS) generation, antioxidant enzyme activity and pro-inflammatory cytokine expression levels. In addition, it would be beneficial to investigate the possible synergistic effects of combining these natural extracts with other known antioxidants or anti-inflammatory agents. This future research will help to validate and expand the application of these sustainable extracts, promoting both health and environmental benefits. In conclusion, the present study contributes to mitigation strategies by providing evidence of the potential of natural extracts from spirulina and fish by-products in reducing CIT toxicity.

## Data Availability

The original contributions presented in the study are included in the article, further inquiries can be directed to the corresponding author.

## Abbreviations and Acronyms

AEGT	aqueous extract of the seaweed *Gracilaria tenuistipitata*
ALP	alkaline phosphatase
ANOVA	Analysis of Variance
ATCC	American Type Culture Collection
C2C12	mouse skeletal muscle
Caco-2/TC7	human colon adenocarcinoma cell line
CIT	Citrinin
DM	Dry matter
DMEM	Dulbecco’s Modified Eagle Medium
DMSO	Dimethyl sulfoxide
EFSA	European Food Safety Authority
FBS	fetal bovine serum
FCP	fish collagen peptides
GOT	glutamic-oxaloacetic transaminase
GPT	glutamic pyruvic transaminase
H1299	human non-small cell lung carcinoma cell line
HaCaT	adult human skin spontaneously transformed aneuploid immortal keratinocyte cell line
HepB3	human hepatocarcinoma cell line
HepG2/CYP2E1	HepG2 cells transfected with human CYP2E1 cDNA
HepG2	human hepatocarcinoma cell line
HPO	Head PEF Optimal extract
HT-29	human colon adenocarcinoma cell line
IC50	Inhibitory Concentration 50%
LDH	lactate dehydrogenase
LPS	lipopolysaccharide
MTT	(3-[4,5-dimethylthiazol-2-yl]-2,5 diphenyl tetrazolium bromide)
nHDF	Normal Human Dermal Fibroblasts
PEF	Pulsed Electric Fields
PI	Propidium Iodide
PK15	porcine kidney epithelial
SEM	Standard Error of the Mean
SH-SY5Y	human neuroblastoma cell line
SpEe	spirulina (*Arthrospira platensis*) ethanolic extract
TDI	Tolerable daily intake
TM4	mouse Sertoli cell line
V79	Chinese hamster lung fibroblasts
